# Plant-Derived Secondary Metabolites Modulating Inflammation-Driven Pathways in Hepatocellular Carcinoma: Preclinical Insights

**DOI:** 10.3390/cimb48020172

**Published:** 2026-02-02

**Authors:** Sergio Arael Mendoza-Calderón, Holanda Isabel Cruz Luis, Laura Pérez-Campos Mayoral, Itzel Patricia Vásquez-Martínez, Eduardo Pérez-Campos, Irma Leticia Bazán Salinas, Juan de Dios Ruiz-Rosado, Nahui Samanta Nájera-Segura, Efrén Emmanuel Jarquín González, Jeanet Elizabeth Aragón Ayala, Christopher Torres Flores, Serafina Pérez Rodríguez, María Teresa Hernández-Huerta, Hector A. Cabrera-Fuentes

**Affiliations:** 1 Centro de Investigación Facultad de Medicina UNAM-UABJO, Facultad de Medicina y Cirugía, Universidad Autónoma “Benito Juárez” de Oaxaca, Oaxaca 68020, Mexico; sergio_arael@outlook.com (S.A.M.-C.); d19161670@itoaxaca.edu.mx (H.I.C.L.); lperezcampos.fmc@uabjo.mx (L.P.-C.M.); vami940508.fmc@uabjo.mx (I.P.V.-M.); basi840313.fmc@uabjo.mx (I.L.B.S.); nahuis.ns@itvalletla.edu.mx (N.S.N.-S.); tofc990309.fmc@uabjo.mx (C.T.F.); pers860105.fmc@uabjo.mx (S.P.R.); 2 División de Estudios de Posgrado e Investigación, Tecnológico Nacional de México/IT Oaxaca, Oaxaca de Juárez 68030, Mexico; pcampos@itoaxaca.edu.mx (E.P.-C.); jeanet_aragon@hotmail.com (J.E.A.A.); 3 Clinical Pathology Laboratory, “Eduardo Pérez Ortega”, Oaxaca 68000, Mexico; 4 Kidney and Urinary Tract Research Center, Abigail Wexner Research Institute, Nationwide Children’s Hospital, Columbus, OH 43215, USA; juandedios.ruizrosado@nationwidechildrens.org; 5 Division of Nephrology and Hypertension, Nationwide Children’s Hospital, Columbus, OH 43205, USA; 6 División de Estudios de Posgrado e Investigación, Tecnológico Nacional de México, Instituto Tecnológico del Valle de Etla, Santiago Suchilquitongo, Oaxaca 68030, Mexico; 7 Dirección General de los Servicios de Salud de Oaxaca, Secretaria de Salud, Servicios de Salud de Oaxaca, Oaxaca 68000, Mexico; drefrenjg@icloud.com; 8 Secretaría de Ciencia, Humanidades, Tecnología e Innovación (SECIHTI), Faculty of Medicine and Surgery, Autonomous University “Benito Juárez” of Oaxaca, Oaxaca 68020, Mexico; 9 División de Investigación y Desarrollo Científico, Benemérita Universidad de Oaxaca, Oaxaca 68000, Mexico; 10 R&D Group, Vice Presidency Scientific Research & Innovation, Imam Abdulrahman bin Faisal University (IAU), Dammam 31451, Saudi Arabia

**Keywords:** hepatocellular carcinoma, secondary metabolites, plant-derived compounds, anticancer therapy, molecular signaling pathways

## Abstract

Hepatocellular carcinoma (HCC) is a leading cause of cancer-related mortality worldwide, primarily driven by chronic inflammation from viral hepatitis, metabolic dysfunction, alcohol-induced liver disease, and cirrhosis. Conventional therapies often fail in advanced stages, highlighting the need for mechanism-based, precision-guided interventions. Plant-derived secondary metabolites represent a promising class of bioactive compounds with structural diversity, multitarget activity, anti-inflammatory effects, and favorable toxicity profiles. This review follows a semi-systematic narrative that synthesizes preclinical and experimental evidence on the anti-inflammatory and anticancer properties of key phytochemicals, including epigallocatechin-3-gallate, galangin, resveratrol, quercetin, curcumin, berberine, genistein, and thymoquinone. These compounds consistently modulate critical inflammation-driven signaling pathways, PI3K/AKT/mTOR, NF-κB, JAK/STAT, Wnt/β-catenin, and MAPK, resulting in apoptosis induction, cell cycle arrest, inhibition of angiogenesis, and reduced invasion and metastasis in multiple HCC models. Despite strong preclinical evidence, clinical translation remains limited by variable bioavailability, incomplete safety data, and insufficient human studies. A staged development strategy is recommended: standardized formulations, Good Laboratory Practice-compliant pharmacokinetic/toxicology studies, validation in patient-derived models, and early-phase, biomarker-guided clinical trials with combination therapy arms. Addressing regulatory, manufacturing, and quality control considerations will be essential for advancing these compounds as adjuvant or complementary agents in precision HCC therapy.

## 1. Introduction

Hepatocellular carcinoma (HCC) is the most common cause of liver cancer and the second leading cause of cancer-related death globally [1,2]. In 2022, more than 865,835 primary liver cancer cases and 758,444 deaths [3,4] occurred, with HCC accounting for approximately 80% and 90% of all primary liver cancers [5]. Several risk factors for developing HCC, the leading causes are viral hepatitis (B and/or C), alcohol abuse, obesity, consumption of foods infected with the fungal toxin-aflatoxin B1 (AFB1), liver disease, non-alcoholic fatty liver disease, diabetes, obesity and smoking [6,7]. HCC has a survival rate that does not exceed 20% between the 3-year [8] and 5-year [9], and only approximately 15% patients are eligible for the potentially curative treatments [8,10].

At present, the systemic and locoregional treatments most used in patients with HCC include liver transplantation, surgical resection, radiofrequency or microwave ablation, transarterial chemoembolization, and systemic chemotherapy [11,12]. Liver resection or transplantation, radiofrequency ablation are potentially curative therapies [10]. These treatments are usually effective when the tumor is in an early stage of development, but are ineffective in advanced stages of the disease, and also produce side effects that detract from patients’ quality of life. It is essential to identify new treatment modalities that modulate the mechanisms of abnormal signal transduction pathways in cancer and their effects on tumorigenesis, apoptosis, and metastasis, while minimizing secondary effects [13].

Medicinal plants and herbs have been utilized in traditional medicine since prehistoric times. Today, they are still a vital source of active ingredients in many medications. Plant-based metabolites exhibit a range of beneficial effects in humans, including analgesic, antibacterial, anti-hepatotoxic, antioxidant, antitumor, and immunostimulating properties, among others [9,14]. Therefore, we aimed to review the anticancer potential of plant-derived secondary metabolites in hepatocellular carcinoma, highlighting their effects on molecular mechanisms, inflammation-associated signaling pathways, and cellular outcomes, including apoptosis, proliferation, angiogenesis, invasion, and metastasis.

**Figure 1:**
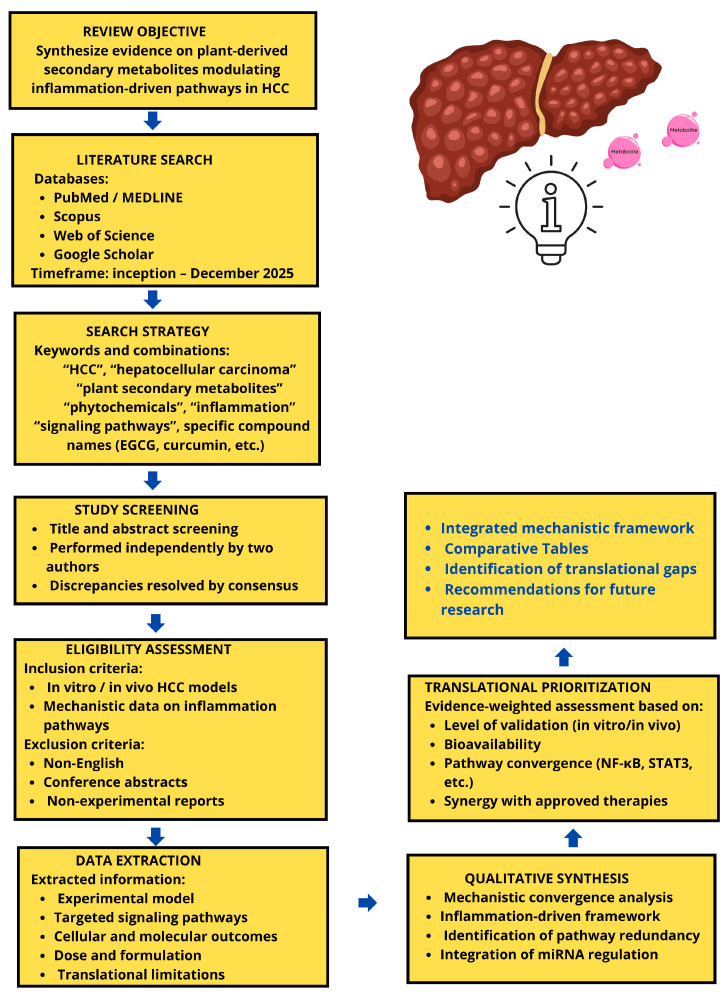
Methodological workflow of the semi-systematic narrative review. This figure summarizes the methodological approach used to identify, screen, and synthesize evidence on plant-derived secondary metabolites targeting inflammation-driven pathways in hepatocellular carcinoma. A structured literature search was conducted across multiple databases up to December 2025. Studies were screened independently by two authors using predefined inclusion and exclusion criteria. Data were qualitatively synthesized with emphasis on mechanistic convergence, inflammation-associated signaling, and translational relevance, leading to an evidence-weighted prioritization of candidate compounds.

This review follows a semi-systematic narrative approach to identify relevant experimental, preclinical, and clinical studies of plant-derived secondary metabolites in HCC, we searched PubMed/MEDLINE, Scopus, Web of Science, and Google Scholar for articles published up to December 2025 using combinations of terms including “hepatocellular carcinoma”, “HCC”, “plant secondary metabolites”, “phytochemicals”, “epigallocatechin-3-gallate”, “resveratrol”, “quercetin”, “berberine”, “genistein”, “curcumin”, “inflammation”, and “signaling pathways”. Additional sources were identified by screening reference lists of relevant articles and reviews (Figure 1). We included original research reporting molecular mechanisms, in vitro or in vivo efficacy, pharmacokinetics, or safety data for plant secondary metabolites in HCC models; we excluded non-English items, conference abstracts without full data, and articles lacking primary experimental results. Two authors independently screened titles and abstracts and reviewed full texts when necessary; disagreements were resolved by consensus. The studies identified through this targeted, narrative search are synthesized below with an emphasis on inflammation-driven signaling pathways and the anticancer effects of plant-derived secondary metabolites in HCC. The following section provides an overview of HCC epidemiology, risk factors, and clinical challenges to contextualize the therapeutic rationale.

## 2. Sequential Development of HCC

**Figure 2:**
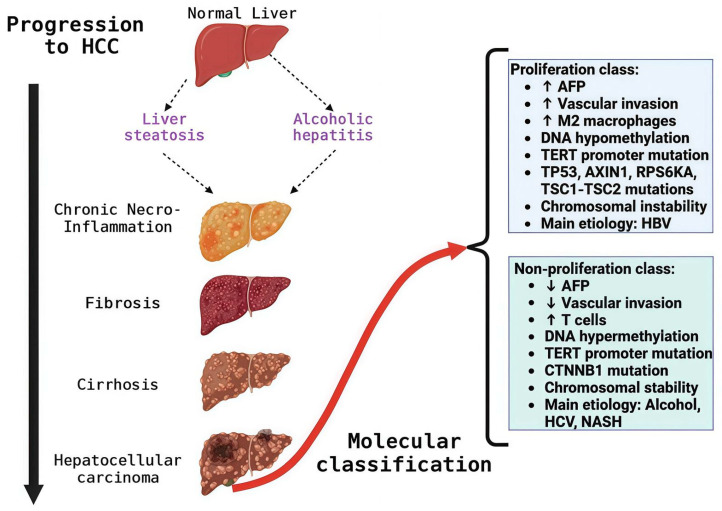
Damage in the liver leads to the development of hepatocellular carcinoma (HCC) and molecular classification. Abbreviations: AFP: α-fetoprotein; HBV: hepatitis B virus; HCV: hepatitis C virus; NASH: non-alcoholic steatohepatitis; HCC: hepatocellular carcinoma; ↑ increases; ↓ decreases.

The progression of HCC is a complex, gradual process (Figure 2) characterized by intermediate cellular changes that ultimately lead to HCC [15,16]. These changes result from genomic and epigenomic alterations that progressively modify the hepatocellular phenotype [17]. Cirrhosis is the most frequent antecedent of HCC, during which nodules are produced that generate an opportune and adequate microenvironment for the mutation of normal hepatocytes to dysplastic hepatocytes, and these to neoplastic lesions until reaching HCC [18].

The sequential development of hepatocarcinogenesis remains incompletely understood. However, clinical and epidemiological studies have revealed that approximately 90% of HCC cases arise from chronic inflammation and tissue damage. This process involves various cellular mechanisms, including oxidative stress, hypoxia, alterations in the tumor microenvironment, chymosin and growth factor activity, cytokine transcription and activation, and DNA damage and methylation [19]. In the pre-malignant environment, inflammatory cells send large amounts of growth factors, cytokines, chemokines, prostaglandins, and proangiogenic factors, which makes the liver environment a favorable site for the survival of hepatocytes altered through the mutation of hepatocytes due to the accumulation of genetic changes, through the activation of antiapoptotic pathways, and the loss of immune surveillance [20].

During HCC, interactions between different pro-inflammatory molecules (interleukin-6 [IL-6], tumor necrosis factor-α [TNF-α]), and anti-inflammatory cytokines (transforming growth factors α and β [TGF-α and TGF-β, respectively]), as well as transcription factors (nuclear factor κB [NF-κB] and signal transducer and activator of transcription 3 [STAT3]), and their signaling pathways, are involved [19,21]. The final step for malignant transformation in hepatocarcinogenesis includes pathways related to angiogenesis and the capacity for migration and invasion [22].

From a molecular perspective, HCC can be classified into two major molecular groups based on transcriptomic phenotypic classes: proliferation and non-proliferation (Figure 1). The proliferation class includes aggressive, poorly differentiated tumors with high vascular invasion and elevated α-fetoprotein (AFP) levels. HBV-associated tumors frequently activate proliferation pathways like PI3K/AKT/mTOR, RAS–MAPK, MET, and IGF. TP53 mutations, high chromosomal instability, and global DNA hypomethylation also characterize this class [23]. In addition, the non-proliferation class includes less aggressive tumors with well to moderate differentiation, low AFP levels, and infrequent vascular invasion. Associated with non-alcoholic steatohepatitis (NASH), alcoholic steatohepatitis, and HCV infection, these tumors exhibit chromosomal stability and frequent TERT promoter and CTNNB1 mutations [23]. HCC can present with different mutations linked to specific pathways (Table 1) [24,25,26]. High levels of heterogeneity are clinically significant and should be considered when providing treatment options to ensure consistent treatment outcomes.

## 3. Different Cell Signaling Pathways Linked to HCC

Current research regarding tumor signal transduction pathways indicates that HCC progression is caused by the irregular activation of some molecules in different signaling pathways that control the cell cycle, proliferation, differentiation, cell survival, and apoptosis causes the progression of HCC [40], some of the signaling pathways in the HCC process are mentioned below.

The PI3/AKT/mTOR signaling pathway is involved in cell growth, metabolism, apoptosis, and the regulation of survival [41]; activation of this pathway occurs in 30–50% during HCC [42]. This pathway is regulated by the homologous phosphatase and tensin tumor suppressor gene (PTEN), the loss of expression of this gene and the positive activation of p-AKT and p-mTOR are related to tumor grade, vascular invasion, and intrahepatic metastasis. This molecular pathway regulates the intracellular signaling of insulin growth factor (IGF) and epidermal growth factor (EGF) receptors, which, through activation of PI3K, PIP3, and AKT, can phosphorylate mTOR and B-cell lymphoma-2 (Bcl-2). In HCC tumors, IGF, EGF, and mTOR expression are increased, and PTEN activity is reduced in 50% of cases [43,44,45,46].

JAK/STAT pathway is activated by growth factors and by different cytokines; it is involved in cell proliferation, differentiation, and apoptosis [47]. It is characterized by rapid response: activated STAT proteins accumulate in the nucleus; within hours, the signal decays, and they are exported to the cytoplasm for the next signaling cycle. This disintegration involves downregulation of receptors, such as JAK proteins, and reduced STAT-mediated transcriptional activity [6,48]. Activated JAK proteins trigger the transcription of SOCS genes, which are part of the negative feedback loop in the JAK/STAT pathway. Dysregulation of pathway inhibitors, particularly SOCS-1 and SS-1 (a JAK-binding protein), has been detected in HCC [49].

The Wnt/β-catenin signaling pathway contributes to cell homeostasis through proliferation, differentiation, motility, and apoptosis [50]. Approximately between 20% and 40% of HCCs show mutations in this pathway [7]. The pathway involves the Wnt ligand, the frizzled receptor, and regulatory proteins, including GSK-3β and β-catenin [51]. Alterations in this molecular pathway are associated with the development of tumors such as HCC. In this process, hepatocytes that exhibit nuclear translocation of beta-catenin have a high capacity for abnormal cell proliferation and a metastatic environment [45].

The primary signal transduction pathway involved in HCC development is Ras/Raf/MAPK, which is commonly implicated in cell proliferation, growth, differentiation, and survival. Signals from membrane-bound tyrosine kinase receptors, such as insulin-like growth factor receptor (IGFR), endothelial growth factor receptor (EGFR), vascular EGFR, c-Met, and Platelet-derived growth factor receptor (PDGFR), are transduced into the cell via the Ras/Raf/MAPK pathway to regulate various cellular functions. Dysregulation of this pathway leads to irregular cellular activities, including increased cell growth, differentiation, and survival, ultimately resulting in cancer [52,53].

### Treatment of HCC

The only treatment option for HCC patients with unresectable tumors or cirrhosis is liver transplantation [54]. After a liver transplant, five-year survival rates can reach 60–70% [55]. Unfortunately, the number of requests for liver transplants exceeds the number of donor organs available [56]. For this treatment, patient selection with HCC must be carefully considered, and the number and size of tumor nodules must be taken into account [57].

In other settings, surgical resection is the first treatment option when HCC is detected early, although it is feasible in <15% of patients, and the 5-year recurrence rate is approximately 70% [58]. Chemotherapy also has undesirable side effects, generally in normal tissues with high proliferative activity; drug resistance may occur, and due to altered liver function, chemotherapy doses are small. HCC is often undetectable until disease progression, and end-stage chemotherapy exerts little effect [59,60,61]. Sorafenib (SOR) is a molecular targeted drug; it is a multikinase inhibitor that is recognized for its efficacy for the treatment of HCC worldwide, but despite advances in molecular therapy with this drug, the prognosis remains poor in advanced HCC, with 5-year survival rates of 3–11%. However, there is no optimal treatment strategy [62]. Consequently, more effective therapeutic agents are being sought to treat HCC that target tumor cells without affecting normal cells and exhibit synergy with chemotherapeutic agents.

## 4. Secondary Metabolites of Plant Origin as Anticancer Agents

The use of plants is one alternative for the treatment of cancer. There are more than 3000 plants with anticancer properties worldwide [63,64], and it has been reported that 50% of all drugs in clinical use are derived from natural products, their derivatives, or their analogs [65]. Plants are a rich source of natural products of great value to humans, whose biological activity arises from the diversity of species and metabolites, as well as from their capacity to synthesize numerous complex molecules [66].

Natural products and their derivatives exhibit structural diversity that confers distinct biological activities, low toxicity, and minimal side effects, which is why they are increasingly used in the development of new anticancer drugs and other compounds [67]. Each year, several secondary metabolites with cytotoxic activity are isolated from plants, providing new avenues for exploring cancer therapies.

The secondary metabolites of plants are mostly organic molecules synthesized by themselves, which are not essential for their growth, development, and reproduction. They have been classified into three main categories based on their biosynthetic pathways: terpenes, phenolic compounds, and nitrogen-containing compounds [68]. Studies have proposed the use of phytochemicals as adjuvant treatments for hepatocellular carcinoma, given their anticancer properties, particularly their capacity to modulate inflammation-associated signaling pathways implicated in hepatocarcinogenesis.

### 4.1. Epigallocatechin-3-Gallate (EGCG)

#### 4.1.1. Primary Mechanistic Evidence in EGCG

Epigallocatechin-3-gallate (EGCG) is the most abundant active catechin found in green tea extract (*Camellia sinensis*) and represents between 50% and 60% of its content [69,70]. In addition, catechins possess antimutagenic, antidiabetic, anti-inflammatory, antibacterial, and antiviral properties [69]. Preclinical studies report that EGCG inhibits HCC progression and induces apoptosis by modulating several pro-apoptotic and anti-apoptotic proteins [70]. Currently, the compound EGCG is used in clinical trials for the treatment of various cancers and other diseases; however, clinical trials have not yet been conducted in patients with HCC [71]. At the mechanistic level, the evidence indicates that EGCG modulates central pathways involved in inflammation, tumor survival, and angiogenesis, including ERK1/2, JAK/STAT, Delta Notch, PI3K/AKT/mTOR, NF-κB and p53, and VEGF [70,72,73,74,75,76]. This evidence positions the previously mentioned pathways as primary mechanisms of action of EGCG on experimental models of HCC.

#### 4.1.2. Indirect Evidence and Secondary Mechanisms in EGCG

Besides its effects on central signaling pathways, EGCG has been shown to inhibit the growth and proliferation of HCC-derived cells by inducing apoptosis, decreasing the expression of cyclooxygenase-2 (COX-2) and Bcl-2, and activating caspase-9 and caspase-3. In addition, suppression of phosphorylation has been reported in the insulin-like growth factor receptor-1 (IGF-1R) and STAT1/STAT3, which are crucial for cell proliferation and survival [70]. Also, EGCG decreased the expression and secretion levels of molecules such as extracellular signal-regulated kinase (ERK), AKT, GSK-3β, reactive oxygen species (ROS), TNF-α, IL-1β, and IL-6 [70,77,78]. However, in most of these studies, the involvement of these pathways is inferred from downstream molecules, so secondary or contextual mechanisms should be considered.

#### 4.1.3. Experimental Models and Pathophysiological Relevance in EGCG

The available evidence on EGCG in HCC comes primarily from preclinical studies, predominantly in vitro models. Although EGCG is currently in clinical trials for various cancers and other diseases, no specific trials have been conducted in patients with HCC, limiting the direct extrapolation of experimental findings to the clinical context of this neoplasm. However, several preclinical studies have demonstrated that EGCG, when combined with chemotherapeutic drugs, increases tumor cell sensitivity to treatment. Simultaneously, can suppress a range of adverse effects caused by chemotherapy, such as gastrointestinal disorders, nephrotoxicity, and cardiotoxicity, through its anti-inflammatory and antioxidant effects, and thereby improve patients’ quality of life [79].

At the experimental clinical level, nanomodification of EGCG has been shown to significantly enhance its antitumor activity. For example, in combination with doxorubicin (DOX) [70,79,80], where EGCG enhances antitumor activity by inhibiting carbonyl reductase 1 (CBR1) and P-glycoprotein (P-gp) in liver cells, interfering with the metabolic conversion of daunorubicinol (DNROL) to DOX and reducing associated cardiotoxicity [70]. Similarly, the combination of EGCG and sulforaphane with cisplatin has demonstrated greater efficacy in both sensitive and resistant ovarian cancer cells by decreasing cell survival, inducing apoptosis, and blocking the cell cycle in the G2/M phase in a time- and dose-dependent manner [70,81]. Additionally, EGCG has been shown to modulate key signaling pathways in various cancers, including reducing EGFR phosphorylation, suppressing AKT signaling, and potentiating apoptosis induced by hormonal modulators such as raloxifene and tamoxifen [70,82]. In animal models, combination therapy with EGCG has resulted in a marked reduction in tumor volume and weight by inhibiting mTOR and EGFR [70,83,84]. Beyond its direct antitumor effect, EGCG has demonstrated cytoprotective properties in radiation-induced injury models, prolonging the survival of irradiated animals and preserving the integrity of the intestinal mucosa. These effects are associated with activation of the Nrf2 pathway, reduced ROS levels, and inhibition of apoptosis and ferroptosis. Then, these findings support the potential of EGCG as an adjuvant to chemotherapy in HCC [79].

#### 4.1.4. Dose, Bioavailability, and Translational Barriers in EGCG

Although EGCG has shown considerable promise as a therapeutic agent for cancer by targeting multiple proteins involved in tumor growth, survival, and therapeutic resistance, its clinical application is hindered by poor bioavailability. EGCG exhibits high binding affinity in the nanomolar (nM) range toward several molecular targets with established antitumor relevance, including NAD kinase (3 nM), cannabinoid receptor 1 (4 nM), disintegrin and metalloproteinase domain 17 (ADAM17; 4 nM), peptidyl-prolyl cis–trans isomerase (4 nM), the anti-apoptotic protein Bcl-2 (335 nM), and the serine/threonine kinase mTOR (320 nM). Despite these favorable target affinities, achieving therapeutically relevant systemic and intratumoral concentrations of EGCG in vivo remains challenging [70]. However, its health-promoting properties are limited due to its low bioavailability, primarily because of its high instability, limited gastrointestinal tract penetration under specific pH conditions, and rapid clearance [85,86]. It is worth noting that clinical trials have been conducted in which pharmacokinetic parameters were controlled [87], and the results of these studies suggest that adjustments to various parameters could improve EGCG bioavailability. In this context, EGCG encapsulation in nanoparticulate systems (e.g., chitosan and peptides) effectively enhances its intestinal absorption, thereby improving its bioavailability [88].

### 4.2. Galangin

#### 4.2.1. Primary Mechanistic Evidence in Galangin

Galangin (GA, 3, 5, 7-trihydroxyflavone) is a polyphenolic compound extracted mainly from the roots of *Alpinia officinarum* Hance, *Alnus pendula* Matsum, *Plantago major* L., and *Scutellaria galericulata* L. (*S. scrodifolia* Fisch.) [89,90]. Various studies have demonstrated that GA suppresses the growth, proliferation, and viability of HCC [91]. The most consistently described mechanism involves the induction of mitochondrial apoptosis via activation of caspases 3, 8, and 9 in HCC [92,93,94], and identifies caspase-3-dependent apoptosis on PTEN expression as the primary mechanism by which GA mediates apoptosis [95]. The most consistently described mechanism involves the induction of mitochondrial apoptosis via activation of caspases 3, 8, and 9 in HCC [92,93,94], and identifies caspase-3-dependent apoptosis on PTEN expression as the primary mechanism by which GA mediates apoptosis [95,96]. Finally, treatment with GA induced apoptosis by translocating the pro-apoptotic protein Bax to the mitochondria, which released apoptosis-inducing factor and cytochrome c into the cytosol [97].

#### 4.2.2. Indirect Evidence and Secondary Mechanisms in Galangin

In addition, GA has been reported to suppress HCC proliferation by reversing the Warburg effect, decreasing glucose uptake in HCC cells, and suppressing glycolytic processes. This effect is associated with upregulation of pyruvate kinase (PK) and regulation of proteins involved in the glycolytic pathway [92,93]. Although these metabolic changes correlate with inhibition of tumor cell proliferation, a direct causal link between metabolic reprogramming and apoptosis induction has not been fully established [98]. Also, these alterations are driven by oncogenes and tumor suppressor genes that rewire cellular metabolism to meet the demands of rapid cell division, resist cell death, and adapt to the hypoxic conditions within the tumor microenvironment (TME) [98,99]. Therefore, these effects should be regarded as secondary or complementary mechanisms.

#### 4.2.3. Experimental Models and Pathophysiological Relevance in Galangin

The available evidence on GA in HCC derives exclusively from in vitro preclinical studies, mainly using HepG2, Huh7, Hep3B, and PLC/PRF/5 cell lines [100]. In these models, GA arrested the cell cycle in the G0/G1 phase, induced apoptosis without cytotoxicity in normal cells, and, in some cases, displayed protective effects [101,102,103]. However, the lack of in vivo validation and the absence of models incorporating TME factors, chronic inflammation, or immune interactions substantially limit the pathophysiological relevance of the current findings [100,104]. Furthermore, the mechanisms of GA must be studied in clinical trials for it to be considered as a future anticancer drug [105].

#### 4.2.4. Dose, Bioavailability, and Translational Barriers in Galangin

GA has been evaluated at variable experimental concentrations, but there is currently no information regarding its pharmacokinetics, bioavailability, metabolism, or tissue distribution. Due to its molecular mechanism and the lack of cytotoxic effects in normal cells, it is credited with significant benefits in the prevention and treatment of HCC It has come to be considered a promising chemotherapeutic drug [97,106]. Thus, the absence of pharmacological and toxicological data represents a major translational barrier, preventing assessment of its feasibility for clinical development [107].

### 4.3. Resveratrol

#### 4.3.1. Primary Mechanistic Evidence in Resveratrol

Resveratrol (3, 5, 40-trihydroxystilbene) is a compound of the stilbene group that contains phytoalexin, belongs to the polyphenol family, and is found naturally in more than 70 species of plants, some examples are *Polygonum cuspidatum*, *Mulberry* (*Morus* sp.), in grapes and in red wine [108]. Several studies have demonstrated that resveratrol modulates the expression levels of various cyclins and cyclin-dependent kinases (CDKs) and upregulation of tumor suppressor proteins such as p53 and p21, all of which trigger cell-cycle arrest and subsequent induction of apoptosis in cancer cells [109]. Also, resveratrol activates mitochondria and intrinsic components of the apoptotic pathway by releasing cytochrome c and Smac/diablo proteins, resulting in tumor cell death [10,110]. Resveratrol is reported to reduce elevated MLCK (myosin light chain kinase) expression, associated with proliferation and anti-apoptotic effects in DENA-induced HCC in an in vivo model of DENA-induced HCC, suggesting an antitumorigenic mechanism mediated by MLCK downregulation [94]. Further downregulated p-AKT expression and induced PTEN mRNA expression via inhibition of important transcription factors, p-STAT3, VEGF and Bcl2, an antiapoptotic protein [109].

#### 4.3.2. Indirect Evidence and Secondary Mechanisms in Resveratrol

Beyond its direct pro-apoptotic activity, resveratrol exerts indirect anticancer effects through the modulation of biochemical and molecular markers associated with hepatocellular carcinoma progression. In HCC cells, it is associated with apoptosis and reduced hexokinase 2 (HK2) expression, thus HK2 depletion inhibits glycolysis and induces oxidative phosphorylation and leads to decline in ATP supply [111]. Experimental evidence indicates that resveratrol treatment attenuates hepatic tumor nodule formation and significantly reduces serum levels of clinical biomarkers such as α-fetoprotein (AFP), lactate dehydrogenase (LDH), phosphatases, and aminotransferases, reflecting an improvement in liver function and tumor burden. At the molecular level, immunoblot analyzes demonstrate upregulation of p53, enhanced PARP cleavage, mitochondrial release of cytochrome c, and activation of caspase-3, collectively supporting the induction of apoptosis. Although these events are often interpreted as primary antitumor mechanisms, they may also represent secondary consequences of broader metabolic and inflammatory modulation driven by resveratrol. Given that AFP elevation is a hallmark of early HCC and correlates with disease progression, the ability of resveratrol to prevent AFP upregulation in DENA-induced HCC models suggests an indirect regulatory role on tumor-associated metabolic and transcriptional networks, rather than a direct effect on AFP gene expression alone [109,112].

#### 4.3.3. Experimental Models and Pathophysiological Relevance in Resveratrol

The evidence includes in vitro (HCC cells) and in vivo (DENA-induced HCC rats) and indicates that in cellular systems, resveratrol modulates cell-cycle regulators, cyclins, CDKs, and tumor suppressor proteins (p53, p21), leading to cell-cycle arrest and apoptosis through activation of the intrinsic mitochondrial pathway. In vivo, resveratrol treatment attenuates hepatic tumor nodule formation and reduces serum biomarkers associated with HCC progression and liver injury, including AFP, LDH, phosphatases, and aminotransferases, indicating reduced tumor burden and improved hepatic function. Molecular analyses in these models reveal upregulation of apoptotic markers, including p53, PARP cleavage, cytochrome c release, and caspase-3 activation [94,108,109,110]. However, while these findings demonstrate consistent antitumor effects across experimental systems, they are largely obtained in simplified carcinogenesis models that do not fully capture the complexity of human HCC, including tumor heterogeneity, immune interactions, and chronic inflammatory microenvironments. Consequently, although the models employed provide mechanistic and phenotypic relevance for preclinical evaluation, their pathophysiological translatability to human HCC remains limited.

#### 4.3.4. Dose, Bioavailability, and Translational Barriers in Resveratrol

Studies indicate that resveratrol effectively inhibits DEN-induced hepatocellular carcinoma and significantly increases apoptotic tumor cells, particularly at doses around 100 mg/kg, supporting its antitumor activity in preclinical models. Across the literature, however, resveratrol has been administered over a wide and heterogeneous dose range, spanning micromolar concentrations [µM] in vitro (0–200 µM) and systemic doses in vivo (10–300 mg/kg/day) depending on the experimental design. In chemically induced HCC models in rats, antitumor and anti-inflammatory effects were most consistently observed at doses between 20 and 100 mg/kg/day, whereas higher doses (up to 300 mg/kg/day) were required in some studies to suppress inflammatory cytokines and oxidative stress markers. In contrast, many in vitro studies used concentrations ≥50 µM to induce apoptosis, cell-cycle arrest, or pathway inhibition, raising concerns about physiological relevance given resveratrol’s known bioavailability limitations [96,112].

Importantly, dose–response relationships were rarely systematically explored, and similar molecular endpoints were achieved with markedly different dosing regimens. Together, these observations highlight a critical translational gap: although resveratrol shows promise in preclinical HCC models, further studies, particularly in humans, are required to define optimal dosing, treatment duration, formulation strategies, and safety profiles before clinical applicability can be realistically assessed. Thus, chemopreventive and chemotherapeutic properties have been described across the three stages of carcinogenesis; consequently, they can be considered therapeutic options for different types of cancer [113,114].

### 4.4. Quercetin

#### 4.4.1. Primary Mechanistic Evidence in Quercetin

Quercetin (3, 3′, 4′, 5, 7-pentahydroxyflavone) is a flavonoid compound that is abundantly present in various fruits, vegetables, and cereals [115]. Quercetin-induced cell apoptosis involves G2/M arrest, apoptosis, and cell death and is associated with increased expression of p53 and p21; decreased levels of cyclin D1, cyclin-dependent kinase (CDK) 2, and CDK7; and the generation of reactive oxygen species [116,117]. It also contributes to the regulation of survival/proliferation (AKT, ERK) and death signaling (caspase-3, p38, and the imbalance of proapoptotic and antiapoptotic proteins, including Bcl-2) in cell lines such as HepG2 [118,119].

#### 4.4.2. Indirect Evidence and Secondary Mechanisms in Quercetin

In addition to its direct cytotoxic effects, quercetin exerts multiple indirect anticancer actions that contribute to its overall antitumor efficacy [120,121]. Quercetin displays pronounced antioxidant and anti-inflammatory properties, reducing oxidative stress and inflammatory responses associated with chronic liver injury, particularly in early stages of hepatocarcinogenesis [122,123]. It has also been shown to inactivate hepatic stellate cells, key mediators of liver fibrosis and inflammation, by modulating their polarization state, thereby limiting the formation of a pro-tumorigenic fibrotic microenvironment. A study evaluated the effects of quercetin on cell migration and invasiveness, reporting that this flavonoid regulated the expression of epithelial and mesenchymal markers, thereby inhibiting epithelial-to-mesenchymal transition (EMT) and invasiveness in LM3 CHC cells [124]. These effects, while not always directly linked to apoptosis induction, contribute to secondary mechanisms that restrain tumor progression, invasion, and microenvironment remodeling.

#### 4.4.3. Experimental Models and Pathophysiological Relevance in Quercetin

Some research has investigated synergistic combinations of quercetin to enhance efficacy against liver cancer. In several cases, the properties of the first-line drug sorafenib (SOR), were demonstrated in HCC cell lines (HepG2, HuH7, and Hep3B2) by reducing their maximum mean inhibitory concentration (IC_50_) and enhancing their tumor-suppressing activity [125,126]. These models consistently show that quercetin reduces proliferation, induces apoptosis, arrests the cell cycle, and inhibits migration and invasiveness. Importantly, quercetin has also been evaluated in combination strategies with SOR, the first-line systemic therapy for advanced HCC. In these studies, quercetin significantly reduced the IC_50_ of SOR and enhanced its tumor-suppressive activity, suggesting biological relevance in a therapeutic context. The synergistic effects observed may involve modulation of circ_SPECC1, hsa-let-7c-5p, cell-cycle regulation, and JAK–STAT signaling pathways [127]. While these findings support the pathophysiological relevance of quercetin, most evidence remains confined to simplified experimental systems, highlighting the need for validation in in vivo and clinically relevant models.

#### 4.4.4. Dose, Bioavailability, and Translational Barriers in Quercetin

Quercetin can inactivate liver stellate cells, which are key to the development of chronic liver injury and the generation of an inflammatory and fibrous microenvironment. This flavonoid modulates its polarization, thereby restricting alterations in liver cells [128]. It has been shown to induce apoptotic cell death by regulating the cell cycle and suppressing anti-apoptotic proteins, including the regulatory protein Sp1 [116]. Quercetin inhibits PI3K-AKT-mTOR signaling in vitro at 0.5–100 µM concentrations and 50 mg/kg doses in vivo [129]. But with its promising anticancer effects, this toxicological profile reveals dose-dependent hepatotoxicity, nephrotoxicity, and potential drug interactions due to cytochrome P450 enzyme inhibition [129].

Then, despite its promising anticancer properties, the clinical translation of quercetin is limited by pharmacokinetic challenges, particularly its low oral bioavailability, rapid metabolism, and limited systemic exposure. Experimental studies employ a wide range of concentrations, complicating dose extrapolation to humans. Furthermore, differences in formulation, purity, and delivery routes across studies hinder direct comparison of efficacy. Although synergistic effects with SO suggest potential utility as an adjuvant agent, comprehensive pharmacokinetic, toxicological, and dose-optimization studies are required [123,125]. Overcoming these barriers through formulation strategies such as nanoencapsulation or combination delivery systems will be critical for advancing quercetin toward clinical application in hepatocellular carcinoma. These studies make quercetin a promising agent for improving outcomes in the therapy of liver pathologies.

### 4.5. Others

Several phytochemicals exhibit context- and dose-dependent effects. For example, capsaicin may exert pro-oxidant or pro-tumorigenic effects at low doses, and resveratrol responses depend on p53 status. These inconsistencies warrant cautious interpretation.

Oroxylin A (C16H12O5) is a natural monoflavonoid extracted from the root of Scutellaria baicalensis Georgi or S. radix, manifests as a bioactive flavone, and exists in the form of aglycone. A exhibits multiple biological activities relevant to HCC, including antiangiogenic effects [130], restores liver function [131], and exerts chemopreventive activity in HCC. At the cellular level, Oroxylin A and induces apoptosis through the suppression of the expression of the Bcl-2 protein, the pro-caspase-3 protein, and the increase in the number of apoptotic cells, as well as G2/M phase arrest [132,133]. In addition, it has been reported that Oroxylin A regulates tumor glycolytic metabolism by inhibiting MDM2 transcription, leading to the stabilization of p53 without directly affecting its transcription. This regulatory effect is mediated through the SIRT3–PTEN–MDM2 signaling axis, demonstrates efficacy both in vitro and in vivo, proposes a p53-independent mechanism that is also applicable to p53-mutant cells, and provides a strong mechanistic and translational rationale for future clinical development [134].

Berberine (BBR) is an alkaloid isolated from the Coptidis rhizome. It exhibits anticancer activity in human HCC cell lines by inducing apoptosis and inhibiting tumor cell proliferation [135]. At the molecular, BBR induce apoptosis in HepG2 cells is associated with CD147 downregulation [136]. In parallel, BBR induces apoptosis in HCC cells primarily through caspase-dependent mitochondrial pathways mediated by AMPK activation. In HepG2 modulates the p53 signaling pathway by suppressing MDM2, the endogenous inhibitor of p53, at the post-transcriptional level, thereby regulating p53 expression and activity [137]. BBB demonstrates synergistic antitumor activity in combination therapies. The combination of BBB and vincristine showed synergistic pro-apoptotic activity against hepatoma cell lines [138]. Moreover, clinical studies suggest that combining SOR with BBR not only enhances the proliferation-inhibitory effects, apoptosis, and reactive oxygen species (ROS) generation induced by SOR but also sensitizes tumor xenograft models to SOR. Notably, this synergistic effect depends on AMPK activation and inhibition of the mTOR signaling pathway in human hepatoma cells [139].

Genistein (4′,5,7-trihydroxyisoflavone) is an isoflavone derived from soy family. It exhibits chemopreventive activities across the stages of carcinogenesis, inhibiting tumor cell proliferation, promoting tumor cell differentiation, and inducing cell cycle arrest and apoptosis in specific cell types [140,141]. At the mechanistic level, genistein activates the mitochondrial apoptosis pathway via caspase-9 and -3, and the Fas pathway by increasing the expression of Fas, FasL, and p53 [142,143]. In addition, genistein-mediated mitochondrial damage by activation of caspase-2, and triggers apoptotic cell death by upregulating TNF-α, FasL, TRADD, and FADD, leading to activation of caspase-8 [144]. Beyond apoptosis, genistein exhibits anti-invasive and antimetastatic activities against TPA-mediated metastasis by downregulating MMP-9 and EGFR, thereby suppressing the transcription factors NF-κB and AP-1 [145]. Using a network pharmacology approach, this study identified multiple therapeutic targets of genistein in hepatocellular carcinoma, highlighting cell-cycle regulation as a central mechanism. Genistein exhibited strong binding affinity for key proteins, including CDC25C and MELK. It inhibited tumor proliferation by inducing G2/M-phase arrest, as validated in vitro and in vivo, providing a solid basis for its potential clinical application in HCC [146].

Atractylenolide II (AT-II) is a natural sesquiterpenoid monomer with antitumor activity. It has been reported to modulate the proliferation, ferroptosis, and immune escape of HCC cells by regulating the TRAF6/NF-κB pathway [147]. In addition, studies have shown that FXR activation by AT-II reduces endoplasmic reticulum (ER) stress, lipogenesis, and inflammation associated with NAFLD, which are key processes in the progression toward hepatocarcinogenesis. These effects position the FXR–SERCA2–eIF2α axis as a therapeutic and chemopreventive target in liver cancer [148].

Lariciresinol (LA) is a lignan and a major active component in many traditional medicinal plants, such as Patrinia, with reported antitumor activity against liver cancer. It exhibits significant cytotoxicity by inhibiting cell proliferation and possibly inducing apoptosis via activation of the mitochondrial-mediated apoptosis pathway [149]. In HepG2 cells, LA inhibited cell proliferation and induced apoptosis by and induced cell cycle arrest in S phase [150], and based on a decrease in ΔΨm (mitochondrial membrane potential); deliver of cytochrome c; activation of caspase-9/-3 and poly(ADP-ribose) polymerase; and a decrease in the proportion of Bcl-2/Bax [149].

Thymoquinone (TQ) was identified as the active component of N. sativa responsible for most of its therapeutic effects [151,152,153]. TQ acts as a potent antioxidant, reducing oxidative stress (malondialdehyde, MDA; lipid peroxidation, LPO; nitric oxide, NO) and restoring the antioxidant system (reduced glutathione, GSH; glutathione peroxidase, GPx; catalase, CAT; glutathione S-transferase, GST). TQ also exhibits a marked anti-inflammatory effect by inhibiting pro-inflammatory cytokines such as TNF-α, IL-1β, and IL-6, and reducing inflammatory mediators such as cyclooxygenase-2 (COX-2), monocyte chemotactic protein-1 (MCP-1), migration inhibitory factor (MIF), and myeloperoxidase (MPO). Furthermore, TQ exerts antifibrotic activity by inhibiting activation of hepatic stellate cells (HSCs) through reduced levels of transforming growth factor beta (TGF-β), smooth muscle alpha-actin (α-SMA), and type I collagen, and by activating AMP-activated protein kinase (AMPK). With respect to apoptosis, TQ modulates it in a context-dependent manner, showing protective effects in damaged liver tissue and promoting apoptosis in tumor cells, partly through inhibition of NF-κB. In HCC, TQ inhibits tumor proliferation by inducing cell cycle arrest at the G1/S transition via increased p21 (a cyclin-dependent kinase inhibitor) and by suppressing oncogenic pathways, including Bcl-2 and the Notch pathway. Finally, TQ has been identified as a safe and multifunctional hepatoprotective agent that reduces liver damage induced by surgery and chemotherapy, thereby improving the tolerability and outcomes of multimodal treatment in patients with HCC [153].

Curcumin is a polyphenolic compound derived from the rhizomes of *Curcuma longa*. It has been shown to exert antitumor effects across various cancers, including hepatocellular carcinoma, by modulating key pathways such as TGF-β, protein kinase B, and caspase-3. At a mechanistic level, it acts by reducing oxidative stress through the decrease in ROS and the activation of antioxidant systems such as GSH and Nrf2, in addition to interfering with the function of hepatic stellate cells, limiting the secretion of pro-angiogenic and pro-tumor factors such as IL-6, VEGF, and SDF-1, and HIF-1α-mediated signaling. Furthermore, curcumin regulates key tumor-survival pathways, including PI3K/AKT/GSK-3β, Wnt/β-catenin, NF-κB, and AMPK, thereby promoting cell-cycle arrest and mitochondrial apoptosis, partly through inhibition of BCLAF1 and modulation of p53. Additionally, it has been shown to induce autophagy and pyroptosis and, in a context-dependent manner, to affect processes such as ferroptosis and cupoptosis. Together, these findings support curcumin as a multi-target agent with antiproliferative, antiangiogenic, pro-apoptotic, and antimetastatic effects in HCC [154,155].

Capsaicin (trans-8-methyl-N-vanillyl-6-nonenamide) is an alkaloid, the most abundant in peppers. Several studies have investigated the functions of capsaicin at various stages of HCC oncogenesis [156]. One of capsaicin’s primary targets is the TRPV1 receptor, whose activation increases intracellular Ca^2+^, triggering signals associated with apoptosis, AMPK activation, and cell-cycle regulation via p53 and p21. At the metabolic and survival levels, capsaicin inhibits the PI3K/AKT/mTOR pathway, promoting apoptosis and autophagy, and modulates pathways including STAT3, EGFR, and NF-κB, thereby reducing proliferation, inflammation, and tumor resistance. It can also induce ER stress, mitochondrial release of cytochrome c, activation of caspase-3, and decrease anti-apoptotic proteins such as Bcl-2, xIAP, and cIAP1. Regarding angiogenesis, capsaicin exhibits anti-angiogenic effects by suppressing VEGF-mediated signaling, limiting endothelial proliferation and differentiation. Furthermore, it regulates oxidative stress bidirectionally: at high concentrations, it induces ROS generation that promotes apoptosis (via NADPH oxidase, ceramides, and TRAIL), whereas at low doses it can favor pro-tumoral processes, highlighting its dose-dependent nature. Finally, its synergy with SOR is noteworthy: the combination enhances antiproliferative, pro-apoptotic, antimetastatic, and anti-angiogenic effects by co-inhibiting PI3K/AKT/mTOR, STAT3, and EMT, suggesting that capsaicin could help overcome SO resistance. Taken together, these data position capsaicin as a promising multi-target agent in HCC, although further studies are needed to define its therapeutic thresholds and clinical safety precisely [157].

Hispidulin is a phenolic flavonoid isolated from the medicinal plant *S. involucrata*, with reported antineoplastic activity [158]. Reports indicate that hispidulin induces apoptosis and reduces cell viability in HCC cells. Mechanistically, its pro-apoptotic effects are partly mediated by activation of ER stress signaling by hispidulin contributes in part to its pro-apoptotic effect on HCC cells, which is also associated with mitochondrial apoptosis and modulation of the AMPK/mTOR signaling pathway [157]. Analysis of 39 studies suggests that hispidulin has therapeutic potential, as it reduces tumor development and inflammation more effectively than oxidative effects via the Nrf2 and HO-1 pathways, while reducing pro-inflammatory markers, including TNF-α, IL-1β, and iNOS, rather than through direct oxidative mechanisms [159].

There is a wide variety of secondary metabolites (Table 2) present in plants that exhibit unique anticancer effects; however, their use in clinical practice is limited by their physicochemical properties, low bioavailability, and/or toxicity. Nevertheless, they can serve as the basis for drug development. Therefore, we emphasize the importance of investigating them, as strategies such as modifying their chemical structures can enhance their anticancer activity and selectivity, improve their absorption, distribution, metabolism, and excretion, and reduce their toxicity and side effects [68].

### 4.6. Bidirectional Interaction Between MicroRNAs and Secondary Metabolites

MicroRNAs (miRNAs) and secondary metabolites maintain a close functional relationship in the regulation of key processes for cell maintenance and survival, particularly in pathological contexts such as inflammation and cancer. Various plant metabolites can alter the expression of specific miRNAs, acting on signaling pathways associated with proliferation, apoptosis, metabolism, and the inflammatory response (Table 2). In turn, miRNAs regulate genes involved in metabolic pathways, influencing the biosynthesis, activity, and cellular response to these bioactive compounds. This bidirectional interaction allows metabolites to exert indirect epigenetic effects by regulating miRNAs, contributing to the modulation of complex molecular networks involved in tumor progression and providing a mechanistic framework for the development of their therapeutic potential.

Although the secondary metabolites discussed above differ markedly in chemical structure and primary molecular targets, a convergent mechanistic pattern becomes evident when their effects are examined in the context of HCC pathogenesis. Many of these compounds influence not only tumor cell–intrinsic processes, such as apoptosis and metabolism, but also inflammation-driven signaling pathways that link chronic liver injury to tumor initiation and progression. To integrate these observations, the following subsection examines inflammation as a unifying mechanistic framework underlying the anticancer activity of plant-derived secondary metabolites in HCC.

### 4.7. Inflammation as a Unifying Mechanistic Framework Underlying Phytochemical Activity in HCC

**Figure 3:**
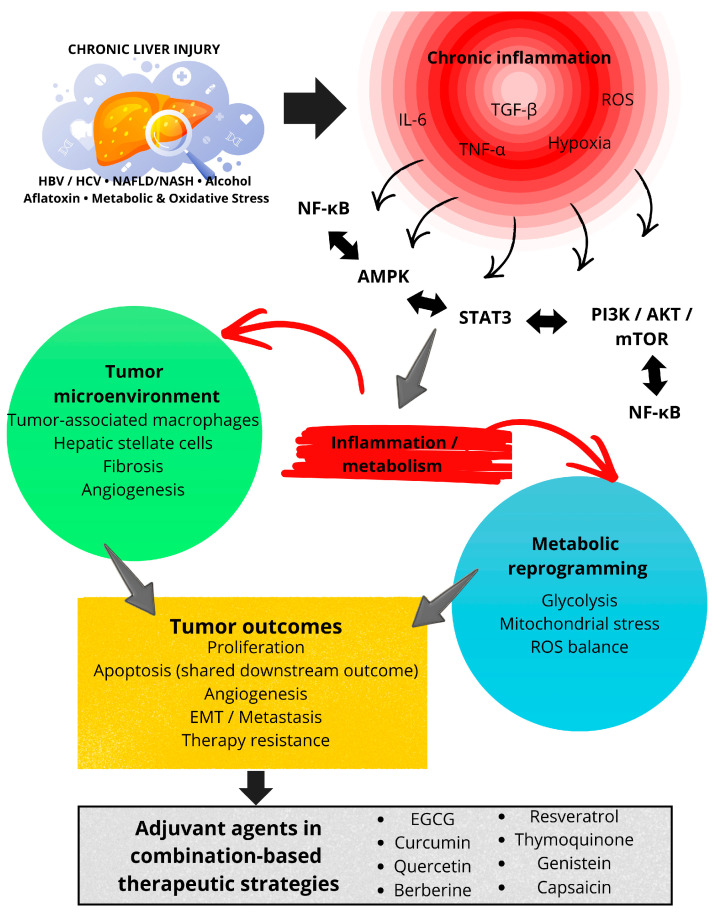
Inflammation-driven signaling network targeted by plant-derived secondary metabolites in HCC. Abbreviations: AMPK, AMP-activated protein kinase; AKT, protein kinase B; EMT, epithelial–mesenchymal transition; EGCG, epigallocatechin-3-gallate; ERK, extracellular signal-regulated kinase; HCC, hepatocellular carcinoma; HBV, hepatitis B virus; HCV, hepatitis C virus; IL-6, interleukin 6; MAPK, mitogen-activated protein kinase; mTOR, mechanistic target of rapamycin; NAFLD, non-alcoholic fatty liver disease; NASH, non-alcoholic steatohepatitis; NF-κB, nuclear factor kappa B; PI3K, phosphoinositide 3-kinase; ROS, reactive oxygen species; STAT3, signal transducer and activator of transcription 3; TGF-β, transforming growth factor beta; TNF-α, tumor necrosis factor alpha.

Chronic inflammation represents a central pathogenic mechanism linking liver injury, fibrosis, and hepatocellular carcinoma development. While individual sections of this review describe the effects of plant-derived secondary metabolites on apoptosis, cell-cycle regulation, and metabolism, these processes are tightly interconnected with inflammation-driven signaling pathways that sustain hepatocarcinogenesis. Therefore, the anticancer activity of phytochemicals should be interpreted within the broader context of inflammation-associated molecular networks. The inflammation-driven signaling network illustrated in Figure 3 provides the conceptual basis for the comparative prioritization framework discussed below.

Figure 3 summarizes inflammation-driven pathway convergence as a unifying mechanistic framework for the anticancer activity of plant-derived secondary metabolites in hepatocellular carcinoma. Rather than acting as isolated cytotoxic agents, phytochemicals converge on central inflammatory and metabolic signaling hubs that integrate chronic liver injury, tumor microenvironment remodeling, and therapeutic resistance.

Accumulating evidence indicates that many plant-derived secondary metabolites modulate inflammatory signaling at multiple levels, including cytokine production, transcription factor activation, immune cell interactions, and stromal remodeling. These anti-inflammatory effects contribute substantially to their antitumor activity in HCC and represent a shared mechanistic feature across structurally diverse compounds.

Importantly, in many studies, pathway modulation is inferred from downstream markers rather than direct assessment of kinase activity or transcriptional regulation. Moreover, pathway crosstalk and compensatory signaling (e.g., ERK or Hippo activation) are rarely explored, despite their relevance in HCC.

#### 4.7.1. Modulation of Inflammatory Cytokine Signaling

Pro-inflammatory cytokines such as IL-6, TNF-α, and TGF-β play a pivotal role in inflammation-driven hepatocarcinogenesis by promoting hepatocyte survival, proliferation, angiogenesis, and immune evasion. Several secondary metabolites discussed in this review, including curcumin, quercetin, thymoquinone, and berberine, have been shown to reduce the expression or activity of these cytokines and their downstream signaling pathways in experimental HCC models [19,21,22,153]. In particular, suppression of the IL-6/STAT3 axis emerges as a recurrent mechanism, as persistent STAT3 activation is a hallmark of chronic liver inflammation and correlates with tumor progression and poor prognosis in HCC [21,23,40].

#### 4.7.2. Regulation of Tumor-Associated Immune Cell Interactions

The inflammatory tumor microenvironment of HCC is characterized by extensive immune cell infiltration, particularly tumor-associated macrophages that often exhibit a pro-tumorigenic M2-like phenotype. Several phytochemicals, including quercetin, curcumin, and thymoquinone, have been reported to modulate macrophage activation and polarization, thereby reducing the production of pro-tumorigenic mediators such as IL-10, TGF-β, and vascular endothelial growth factor [119,153,155]. Through modulation of immune cell–derived inflammatory signals, plant-derived secondary metabolites indirectly influence tumor growth, angiogenesis, and metastatic potential, highlighting their capacity to target both cancer cells and the surrounding inflammatory microenvironment.

#### 4.7.3. Effects on Hepatic Stellate Cells and Inflammation–Fibrosis Coupling

Hepatic stellate cells are key mediators linking chronic inflammation to fibrosis and hepatocarcinogenesis. Activation of these cells contributes to extracellular matrix deposition, cytokine release, and pro-tumorigenic microenvironment remodeling. Compounds such as curcumin and thymoquinone inhibit stellate cell activation by suppressing TGF-β signaling, α-smooth muscle actin expression, and collagen synthesis, thereby attenuating fibrosis-associated inflammatory signaling [119,153,154]. These findings underscore the relevance of phytochemicals in disrupting inflammation–fibrosis coupling, a critical process in the progression from chronic liver disease to HCC.

#### 4.7.4. NF-κB and STAT3 as Central Inflammatory Signaling Nodes Targeted by Phytochemicals

Among inflammation-associated transcription factors, NF-κB and STAT3 serve as central regulators integrating cytokine signaling, oxidative stress, immune responses, and cell survival in hepatocellular carcinoma. Aberrant activation of these pathways is frequently observed in inflammation-driven HCC and contributes to tumor initiation, progression, and therapeutic resistance [19,21,23,40].

Notably, a convergence of evidence indicates that many plant-derived secondary metabolites, including EGCG, curcumin, quercetin, berberine, genistein, capsaicin, and thymoquinone, attenuate NF-κB and/or STAT3 signaling. Inhibition of these pathways provides a mechanistic link between the anti-inflammatory and antitumor effects of phytochemicals, leading to reduced proliferation, enhanced apoptosis, impaired angiogenesis, and suppression of metastatic behavior [72,73,74,75,76,77,78,116,117,124,125,126,127,130,131,140,153,157,168]. Furthermore, suppression of NF-κB- and STAT3-mediated signaling may contribute to the observed synergistic effects between selected phytochemicals and SOR, as these pathways are implicated in resistance to systemic HCC therapies [127,130,131,140,168].

Together, these findings indicate that the translational relevance of plant-derived secondary metabolites in HCC is closely linked to their capacity to modulate inflammation-associated signaling networks, providing a rationale for their comparative prioritization for clinical development. Most studies rely on monoculture HCC cell lines that do not recapitulate tumor microenvironment complexity, immune interactions, or metabolic gradients, limiting translational relevance. Overall, most available data remain preclinical and are frequently derived from simplified in vitro systems. These limitations constrain mechanistic certainty and preclude direct therapeutic extrapolation.

## 5. Comparative Prioritization of Plant-Derived Secondary Metabolites for Clinical Translation in Hepatocellular Carcinoma

Although numerous plant-derived secondary metabolites exhibit anticancer activity in hepatocellular carcinoma, their translational relevance is highly heterogeneous. As highlighted in Section 4, many compounds converge on inflammation-associated signaling pathways; however, demonstration of antiproliferative or pro-apoptotic effects in vitro alone is insufficient to predict clinical utility. Translational potential depends on a convergence of factors, including reproducible in vivo efficacy, pharmacokinetic feasibility, specificity toward inflammation-driven oncogenic signaling, and compatibility with existing therapeutic paradigms. Consequently, a structured prioritization framework is required to distinguish compounds with realistic clinical promise from those of primarily exploratory value.

Based on the evidence synthesized in this review, plant-derived secondary metabolites can be comparatively stratified according to four translationally relevant dimensions: (i) depth and consistency of experimental validation (in vitro versus in vivo versus clinical), (ii) bioavailability and metabolic stability, which remain major barriers for most phytochemicals, (iii) convergence on core inflammation-associated signaling networks central to hepatocarcinogenesis, and (iv) capacity to synergize with approved systemic therapies, particularly multikinase inhibitors such as SOR.

Within this framework, compounds including EGCG, curcumin, quercetin, berberine, resveratrol, and thymoquinone emerge as high-priority translational candidates. These metabolites are distinguished not merely by cytotoxic activity, but by their multilevel interference with inflammation-driven oncogenic circuits, notably NF-κB, STAT3, PI3K/AKT/mTOR, and AMPK signaling pathways, which collectively orchestrate chronic inflammation, metabolic reprogramming, immune evasion, and tumor progression in HCC [11,19,21,22,62]. Importantly, several of these compounds demonstrate efficacy across both in vitro and in vivo models, reinforcing the biological robustness of their antitumor effects.

By contrast, other metabolites such as galangin, lariciresinol, hispidulin, and atractylenolide II, while mechanistically intriguing, are currently supported predominantly by in vitro data or limited animal studies. For these compounds, key translational uncertainties remain, particularly regarding pharmacokinetics, tissue distribution, dose feasibility, and long-term safety, precluding their prioritization for near-term clinical development without further validation.

A critical determinant of translational relevance is pathway convergence. Secondary metabolites capable of simultaneously modulating inflammatory cytokine signaling (IL-6/STAT3), transcriptional regulators of inflammation (NF-κB), and metabolic stress sensors (AMPK–mTOR) are especially compelling, as these interconnected pathways constitute the molecular backbone of inflammation-driven hepatocarcinogenesis [19,21,23,40,41]. Compounds targeting isolated downstream effects without engaging this network are less likely to achieve durable antitumor responses.

In addition, accumulating evidence supports the strategic use of selected phytochemicals as adjuvant agents rather than stand-alone therapies. Quercetin, berberine, capsaicin, and curcumin, in particular, have demonstrated synergistic interactions with SOR, enhancing apoptosis, suppressing angiogenesis, limiting epithelial–mesenchymal transition, and mitigating resistance mechanisms that undermine systemic therapy efficacy [127,130,131,140,157,168]. Such findings underscore the relevance of phytochemicals within combination-based treatment strategies aligned with current clinical practice.

Collectively, these considerations underscore the need to move beyond descriptive inventories of bioactive compounds toward a hierarchized, mechanism-driven prioritization strategy. Prioritization was based on (i) level of experimental validation, (ii) reproducibility, (iii) availability of PK/safety data, and (iv) convergence on inflammation-driven HCC pathways (Table 3). Integrating molecular mechanism, inflammatory context, and translational feasibility is essential for rational experimental design and for accelerating the progression of plant-derived secondary metabolites from experimental observation to clinically actionable interventions in hepatocellular carcinoma.

Although the secondary metabolites discussed above differ in chemical structure and primary molecular targets, a unifying mechanistic theme emerges when their effects are examined in the context of HCC pathogenesis. Many of these compounds converge on inflammation-driven signaling pathways that link chronic liver injury, immune dysregulation, fibrosis, and tumor progression. Therefore, beyond their individual effects on apoptosis, cell-cycle regulation, or metabolism, the anticancer activity of plant-derived secondary metabolites should be interpreted within a broader inflammation-centered framework.

## 6. Limitations of the Review

This review synthesizes predominantly preclinical evidence and therefore has several limitations that should temper interpretation. First, the literature is heavily weighted toward in vitro and animal studies; human clinical data are sparse or absent for most compounds, limiting direct translational inference. Second, substantial heterogeneity across studies in compound source, purity, formulation, dosing, exposure duration, and experimental endpoints impedes quantitative synthesis and meta-analysis. Third, many phytochemicals suffer from poor and variable oral bioavailability; studies employ diverse delivery systems (free compound, extracts, nanoparticles) without standardized pharmacokinetic comparisons, complicating dose extrapolation to humans. Fourth, mechanistic pleiotropy, multitarget effects across signaling networks, makes it difficult to identify primary targets or predictive biomarkers of response. Fifth, safety, toxicology, and drug–drug interaction data (particularly with approved HCC agents such as SOR) are limited and inconsistently reported. Finally, publication and reporting bias likely favor positive findings; negative or null results and adverse signals may be underrepresented. These limitations underscore the need for standardized preclinical protocols, rigorous PK/Tox studies, and biomarker-driven clinical trials.

## 7. Conclusions

Hepatocellular carcinoma is a prototypical inflammation-driven malignancy in which chronic liver injury, fibrosis, metabolic dysregulation, and immune dysfunction converge to promote tumor initiation, progression, and therapeutic resistance. In this context, plant-derived secondary metabolites represent a biologically intriguing class of compounds due to their structural diversity, multitarget activity, and capacity to modulate inflammation-associated signaling pathways central to hepatocarcinogenesis.

Plant-derived secondary metabolites show consistent preclinical activity against inflammation-driven molecular pathways implicated in HCC, including PI3K/AKT/mTOR, NF-κB, JAK/STAT, Wnt/β-catenin, and MAPK signaling. These compounds can induce apoptosis, arrest the cell cycle, inhibit angiogenesis, and reduce invasion/metastasis in multiple HCC models. However, current evidence is insufficient to support routine clinical use due to limited human data, variable bioavailability, and incomplete safety profiling. To advance translation, we recommend a staged strategy: (1) standardize compound characterization and formulations, (2) perform GLP-compliant PK/Tox studies, (3) validate mechanisms in clinically relevant models (patient-derived xenografts, organoids), and (4) design early-phase, biomarker-guided clinical trials that include PK/PD endpoints and combination arms with standard therapies. Addressing manufacturing, regulatory, and quality-control issues for botanical therapeutics will be essential for clinical development. In conclusion, plant-derived secondary metabolites should be viewed not as ready-to-use therapeutics, but as a mechanistically informative and translationally promising resource for targeting inflammation-associated vulnerabilities in hepatocellular carcinoma. Progress toward clinical application will require a cautious, evidence-weighted approach integrating mechanistic rigor, realistic assessment of limitations, and alignment with contemporary combination-based treatment paradigms.

## Figures and Tables

**Table 1:** Mutations and pathways involved in HCC.

Related Pathway	Gen/Target (Expression)	Related miRNAs (Expression)	Study Model
PI3/AKT [27,28]	PTEN	↑ miR-21 miR-23a	In vivo (mice) e in vitro (RAW264.7, mTHP-1 cells)
PI3/AKT and Bcl-2 [29,30]	↑ CDKN1B/p27 ↑ CDKN1C/p57 Bmf	↑ miR-221	In vitro, cell line Hep3B, SNU398, SNU449 In vitro, cell line SNU449, SNU398, Hep3B, HepG2
TGF-*β* [31,32,33]	SMAD4	↑ miR-224	In vitro, cell line HCT116
p53 [34]	Ciclina G	↓ miR-122a	In vitro, cell line HEP3B, SNU449
IRE1*α*/XBP1 [35]	XBP1 Ciclina D	↓ miR-199a	In vitro, cell line Hep3B2.1-7
FXR/SHP [36,37]	↓ SIRT1	↑ miR-34a	In vitro, cell line HepG2
IL-6/STAT3 [38]	G6PC PGC-1*α*	miR-23a	In vivo (mice)
HIH-1a [39]	G6PC	miR-494	In vivo (mice) e in vitro

Abbreviations: PI3/AKT: phosphatidylinositol 3-kinase/protein kinase B; TGF-*β*: Transforming growth factor beta; IRE1*α*/XBP1: Inosito requiring enzyme 1 *α*/X-box-binding protein 1; FXR/SHP: Farnesoid X receptor/Small heterodimer partner; IL-6/STAT3: Interleukin-6/Signal transducers and activators of transcription 3; HIH-1a: Hypoxia-inducible factor 1 alpha; PTEN: Phosphatase and tensin homolog; CDKN1B/p27: Cyclin dependent kinase inhibitor 1B/protein 27; CDKN1C/p57: Cyclin dependent kinase inhibitor 1C/protein 57; Bmf: Bcl-2 modifying factor; SMAD4: Suppressor of Mothers against Decapentaplegic family member 4; SIRT1: Sirtuin 1, G6PC: Glucose-6-phosphatase catalytic; PGC-1*α*: Peroxisome proliferator-activated receptor gamma coactivator 1-alpha; ↑ increases; ↓ decreases.

**Table 2:** Mechanistic convergence and critical appraisal of plant-derived secondary metabolites in hepatocellular carcinoma.

Metabolite	Dominant Mechanistic Category	Main Mechanisms	Related miRNAs	Critical Synthesis
EGCG [15,71,72,73,74,75,76,77,78,85,86,87,88,89,160,161]	Inflammation-driven transcriptional regulation and survival signaling	Modulation of ERK1/2, PI3K/AKT/mTOR, NF-κB, p53, and VEGF; inhibition of COX-2 and Bcl-2; activation of caspases-9/-3; suppression of IGF-1R, ERK, STAT3, Akt, and GSK-3β	↑ miR-548m ↑ miR-194 ↓ miR483-3p ↑ miR16	Preclinical evidence as a modulator of relevant inflammatory pathways in HCC. Evidence based on simplified experimental models. Low bioavailability limits its direct clinical application. Greatest value is translational/adjuvant, not as an immediate clinical therapy.
Galangin [90,91,92,93,94,97,101,102,103,105,106,162]	Mitochondrial apoptosis and metabolic stress	Reversal of the Warburg effect; decreased glucose uptake; regulation of pyruvate kinase and glycolytic enzymes; activation of caspases-3/-8/-9; mitochondrial pathway involving Bax, cytochrome c, and AIF	↓ miR-675	Preclinical in vitro evidence supports mitochondrial pro-apoptotic activity, but absence of in vivo and PK data limits translational potential.
Resveratrol [10,108,113,114,163,164,165,166,167]	Metabolic reprogramming and stress response	Mitochondrial activation (cytochrome c, Smac/Diablo); decreased hexokinase-2; negative regulation of MLCK; cell arrest in face G1 and G2/M, ↑ p21, p53, Bax, iNOS and eNOS expression	↑ miR-143-3p ↑ miR-663 ↓ miR-21	Anti-inflammatory and antioxidant effects that reduce tumor “fitness” rather than directly inducing cell death. High in vitro doses versus low oral bioavailability. Lack of PK/PD optimization and formulation strategies. More plausible as a chemopreventive or adjuvant agent than as monotherapy.
Quercetin [116,117,118,119,120,121,123,124,125,126,127,128,168,169,170,171]	Cell-cycle arrest and microenvironment modulation	Modulation of AKT/ERK; activation of caspase-3 and p38; regulation of Bcl-2; G2/M arrest (↑ p53, p21; ↓ cyclins/CDKs); increased ROS; EMT inhibition; involvement of JAK–STAT and VEGFR/PDGFR pathways. ↓ anti-apoptotic proteins (Sp1 and Sp1 regulatory protein)	↑ miR-122 ↑ miR-34a ↓ miR-21 ↓ miR-27a	Modulation of the fibrotic and inflammatory tumor microenvironment. Low bioavailability and rapid metabolism. Dose-dependent toxicity and CYP-mediated drug interactions. Need for advanced formulations.
Genistein [140,141,142,143,144,145,146,172,173,174,175,176,177]	Cell-cycle control and anti-invasive signaling	Activation of caspases-9/-3 and Fas/FasL pathways; caspase-2 activation; downregulation of MMP-9 and EGFR; inhibition of NF-κB/AP-1; G2/M arrest; strong binding to CDC25C and MELK; ↑ E-cadherin, ↑ -catenin, ↓ N-cadherin, and Vimentin ↓ intrahepatic metastasis due to ↓ EMT, which was correlated with ↓ NFAT1α	↑ miR-1275 ↑ miR-574-3p ↑ miR-34a ↓ miR-223 ↓ miR-21	Reduction in oxidative stress and hepatic inflammation. Modulation of the carcinogenic “soil” (fibrosis, chronic liver damage). Estrogenic and potential endocrine effects. Most plausible role in chemoprevention or adjuvant settings.
Oroxylin A [130,131,132,133,134,178,179,180]	Glycolytic reprogramming and p53 stabilization	Suppression of Bcl-2 and procaspase-3; regulation of glycolytic metabolism via the SIRT3–PTEN–MDM2 axis; stabilization of p53. ↑ Bax protein, ↑ MAC-related mitochondrial apoptosis	↓ miR-221 ↓ miR-155 ↓ miR-21	Reprogramming of tumor glycolytic metabolism. Antiangiogenic and hepatoprotective effects. p53-independent activity strengthens clinical relevance; still lacks clinical PK/PD data.
Berberine [135,136,137,138,139,181,182,183,184,185,186,187]	Energy stress signaling and mitochondrial apoptosis	Caspase-dependent mitochondrial apoptosis via AMPK; CD147 downregulation; regulation of p53/MDM2; mTOR inhibition; increased ROS, Suppression of e NF-kB	↑ miR-23a ↓ miR-21 ↑ miR-122 ↑ miR-146a ↑ miR-22-3p	Inhibits proliferation and enhances ROS-mediated tumor stress. Synergistic with vincristine and SOR via AMPK/mTOR inhibition. Strong adjuvant potential; interactions and dosing need clinical definition.
Atractylenolide II [147,148,188]	Inflammation–metabolism coupling and stress adaptation	Regulation of the TRAF6/NF-κB pathway; activation of the FXR–SERCA2–eIF2α axis; reduction in ER stress, lipogenesis, and inflammation. Ferroptosis and apoptosis.	↓ miR-541-3p	Chemopreventive effects in NAFLD-HCC, reducing ER stress and inflammation. Strong relevance for HCC prevention in metabolic liver disease.
Lariciresinol [149,150,189,190,191,192,193,194,195]	Mitochondrial apoptosis	S-phase cell-cycle arrest; loss of mitochondrial membrane potential; cytochrome c release; activation of caspases-9/-3 and PARP; decreased Bcl-2/Bax ratio	↑ miR-34a ↓ miR-21 ↑ miR-122 ↑ miR-16 ↓ miR-101a	Absence of in vivo and pharmacokinetic validation.
Thymoquinone [151,152,153,196,197,198,199,200,201]	Anti-inflammatory, antioxidant, and antifibrotic regulation	Reduction in MDA, LPO, and NO; increase in GSH, GPx, CAT, and GST; inhibition of TNF-α, IL-1β, IL-6, and COX-2; inhibition of hepatic stellate cells; AMPK activation; G1/S arrest (↑ p21); inhibition of Bcl-2, Notch, and NF-κB	↑ miR-1-3p ↑ miR-877-5p ↑ miR-375 ↑ miR-199a-3p	Potent antioxidant, anti-inflammatory, and anti-fibrotic effects. Strong hepatoprotective activity. Improves tolerability of surgery and chemotherapy; favorable safety profile.
Curcumin [154,155,187,202,203,204,205,206]	Multi-node inflammatory and metabolic modulation	Modulation of PI3K/AKT/GSK-3β, Wnt/β-catenin, NF-κB, and AMPK; reduction in ROS (↑ GSH, Nrf2); inhibition of hepatic stellate cells, VEGF, IL-6, and HIF-1α; mitochondrial apoptosis, autophagy, and pyroptosis	↓ miR-21 ↓ miR-21-5p ↓ miR-130 ↓ miR-221	Anti-fibrotic effects via hepatic stellate cell inhibition. Poor bioavailability; requires advanced delivery systems.
Capsaicin [156,157,207,208]	Stress-activated signaling and angiogenesis inhibition	TRPV1 activation (↑ Ca^2+^); AMPK activation; inhibition of PI3K/AKT/mTOR, STAT3, EGFR, and NF-κB; ER stress; caspase-3 activation; decreased Bcl-2 and IAPs; VEGF suppression; dose-dependent ROS effects	↑ miR-34a	Dose-dependent ROS effects. Synergistic with SOR; may overcome resistance.
Hispidulin [158,159]	ER stress-mediated apoptosis and redox regulation	ER stress; mitochondrial apoptosis; modulation of AMPK/mTOR; activation of Nrf2/HO-1; reduction in TNF-α, IL-1β, and iNOS	---	Strong anti-inflammatory effects. Broad preclinical support; limited in vivo and clinical data.

Abbreviations: HCC: hepatocellular carcinoma; AKT: protein kinase B; AMPK: AMP-activated protein kinase; AIF: apoptosis-inducing factor; AP-1: activator protein 1; Bax: Bcl-2-associated X protein; Bcl-2: B-cell lymphoma 2; CDK: cyclin-dependent kinase; COX-2: cyclooxygenase-2; EGFR: epidermal growth factor receptor; EGCG: epigallocatechin-3-gallate; EMT: epithelial–mesenchymal transition; ERK: extracellular signal-regulated kinase; ER stress: endoplasmic reticulum stress; FADD: Fas-associated death domain protein; FasL: Fas ligand; FXR: farnesoid X receptor; GSK-3β: glycogen synthase kinase 3 beta; GSH: reduced glutathione; GPx: glutathione peroxidase; GST: glutathione S-transferase; HIF-1α: hypoxia-inducible factor 1 alpha; HO-1: heme oxygenase-1; IAPs: inhibitor of apoptosis proteins; IGF-1R: insulin-like growth factor 1 receptor; IL-1β: interleukin-1 beta; IL-6: interleukin-6; iNOS: inducible nitric oxide synthase; JAK–STAT: Janus kinase–signal transducer and activator of transcription; MDM2: mouse double minute 2 homolog; MELK: maternal embryonic leucine zipper kinase; MIF: migration inhibitory factor; MLCK: myosin light chain kinase; MMP-9: matrix metalloproteinase-9; MPO: myeloperoxidase; mTOR: mechanistic target of rapamycin; NF-κB: nuclear factor kappa B; Nrf2: nuclear factor erythroid 2–related factor 2; NO: nitric oxide; PDGFR: platelet-derived growth factor receptor; PI3K: phosphoinositide 3-kinase; PK: pyruvate kinase; p21: cyclin-dependent kinase inhibitor 1A; p38: p38 mitogen-activated protein kinase; p53: tumor protein p53; ROS: reactive oxygen species; SERCA2: sarco/endoplasmic reticulum Ca^2+^-ATPase 2; Smac/DIABLO: second mitochondria-derived activator of caspases; SOR: sorafenib; STAT3: signal transducer and activator of transcription 3; SIRT3: sirtuin 3; TGF-β: transforming growth factor beta; TNF-α: tumor necrosis factor alpha; TRADD: TNF receptor-associated death domain protein; TRAF6: TNF receptor-associated factor 6; TRPV1: transient receptor potential vanilloid 1; VEGF: vascular endothelial growth factor; VEGFR: vascular endothelial growth factor receptor; ↑ increases; ↓ decreases; --- No report.

**Table 3:** Translational prioritization of selected plant-derived secondary metabolites in HCC.

Metabolite	Level of Evidence	Bioavailability Barriers	Key Inflammation-Driven Pathways	Synergy with Approved Therapies	Safety/PK Concerns	Translational Priority	Rationale for Prioritization
EGCG	In vitro + In vivo	High	NF-κB, STAT3, PI3K/AKT	Potential	Hepatotoxicity at high doses	High	Consistent anti-inflammatory effects across models; safety concerns limit dosing
Curcumin	In vitro + In vivo	High	NF-κB, AMPK, Wnt/β-catenin	Yes (SOR)	Poor oral bioavailability	High	Strong mechanistic rationale but severe formulation limitations
Quercetin	In vitro + In vivo	Moderate	AKT/ERK, JAK/STAT	Yes (SOR)	Dose-dependent toxicity	High	Broad pathway targeting but variable reproducibility
Berberine	In vitro + In vivo	Moderate	AMPK, mTOR, NF-κB	Yes (SOR)	Drug–drug interactions (CYP)	High	Metabolic and inflammatory modulation with interaction risks
Resveratrol	In vitro + In vivo	High	STAT 3, NF-κB, metabolic pathways	Potential	Rapid metabolism, low plasma levels	Moderate–High	Pathway convergence but limited systemic exposure
Galangin	In vitro	Moderate	Metabolic reprogramming	Not reported	Poor solubility	Moderate	Exploratory relevance, limited validation
Lariciresinol	In vitro	Unknown	Mitochondrial apoptosis	Not reported	Unknown	Low–Moderate	Limited preclinical evidence suggests anti-inflammatory activity via PI3K/AKT modulation; however, validation is restricted to exploratory in vitro and limited in vivo studies, with insufficient pharmacokinetic data, resulting in low translational priority.
Hispidulin	In vitro	Unknown	ER stress, AMPK/mTOR	Not reported	Unknown	Low–Moderate	Anti-inflammatory and pro-apoptotic effects through NF-κB/STAT3 and ER stress pathways but limited in vivo validation and scarce pharmacokinetic data constrain its translational prioritization.

Abbreviations: AKT, protein kinase B; AMPK, AMP-activated protein kinase; CYP, cytochrome P450; EGCG, epigallocatechin-3-gallate; EMT, epithelial–mesenchymal transition; ER stress, endoplasmic reticulum stress; ERK, extracellular signal-regulated kinase; HCC, hepatocellular carcinoma; IL-6, interleukin 6; MAPK, mitogen-activated protein kinase; mTOR, mechanistic target of rapamycin; NF-κB, nuclear factor kappa B; PI3K, phosphoinositide 3-kinase; PK, pharmacokinetics; ROS, reactive oxygen species; SOR, sorafenib; STAT3, signal transducer and activator of transcription 3; TGF-β, transforming growth factor beta; TNF-α, tumor necrosis factor alpha.

## Data Availability

No new data were created or analyzed in this study. Data sharing is not applicable to this article.

## Abbreviations

α-AFP	α-fetoprotein
AFB1	Aflatoxin B1
AMPK	AMP-activated protein kinase
BBR	Berberine
Bax	Bcl-2-associated X protein
Bcl-2	B-cell lymphoma 2
cox2	Cyclooxygenase-2
DENA	Diethylnitrosamine
EGCG	Epigallocatechin-3-gallate
EMT	Epithelial-to-mesenchymal transition
ERK1/2	Extracellular signal-regulated kinase 1/2
FXR/SHP	Farnesoid X receptor/Small heterodimer partner
G6PC	Glucose-6-phosphatase catalytic subunit
GSK-3β	Glycogen synthase kinase-3 beta
GLP	Good laboratory practice
HCC	Hepatocellular carcinoma
HBV	Hepatitis B virus
HCV	Hepatitis C virus
HIF-1α	Hypoxia-inducible factor 1 alpha
IL-6	Interleukin-6
JAK/STAT	Janus kinase/Signal transducers and activators of transcription
MAPK	Mitogen-activated protein kinase
MDM2	Mouse double minute 2 homolog
MLCK	Myosin light chain kinase
NASH	Non-alcoholic steatohepatitis
NF-κB	Nuclear factor kappa B
PDGFR	Platelet-derived growth factor receptor
PI3K/AKT/mTOR	Phosphatidylinositol 3-kinase/Protein kinase B/Mechanistic target of rapamycin
PK	Pyruvate kinase
PK/PD	Pharmacokinetics/Pharmacodynamics
PGC-1α	Peroxisome proliferator-activated receptor gamma coactivator 1-alpha
PRISMA	Preferred reporting items for systematic reviews and meta-analyses
PTEN	Phosphatase and tensin homolog
ROS	Reactive oxygen species
SIRT1	Sirtuin 1
SOR	Sorafenib
STAT3	Signal transducer and activator of transcription 3
TGF-β	Transforming growth factor beta
VEGF	Vascular endothelial growth factor
Wnt/β-catenin	Wnt/Beta-catenin signaling
